# Advancements in Microwave Ablation Techniques for Managing Pancreatic Lesions

**DOI:** 10.3390/life13112162

**Published:** 2023-11-04

**Authors:** Devarshi R. Ardeshna, Matthew Leupold, Zobeida Cruz-Monserrate, Timothy M. Pawlik, Jordan M. Cloyd, Aslam Ejaz, Hamza Shah, Jordan Burlen, Somashekar G. Krishna

**Affiliations:** 1 Division of Gastroenterology, Hepatology and Nutrition, The Ohio State University Wexner Medical Center, Columbus, OH 43210, USA; 2 Department of Internal Medicine, Ohio State University Wexner Medical Center, Columbus, OH 43210, USA; 3 The James Comprehensive Cancer Center, The Ohio State University Wexner Medical Center, Columbus, OH 43210, USA; 4 Department of Surgery, The Ohio State University Wexner Medical Center, Columbus, OH 43210, USA

**Keywords:** microwave ablation, thermal ablation, pancreatic cancer, neuroendocrine tumors, pancreatic cystic lesions, pancreas tumors

## Abstract

Thermal ablation, including microwave ablation, has become increasingly important in the management of many solid tumors, including primary and metastatic tumors of the liver, kidney, and lung. However, its adoption to treat pancreatic lesions has been slowed due to concerns about potential adverse events. The success of radiofrequency ablation (RFA) in inoperable pancreatic cancers paved the way for its use in pancreatic neuroendocrine tumors and pancreatic cystic neoplasms (PCLs). In the last decade, other thermal ablation techniques, like microwave ablation, have emerged as alternatives to RFA. Microwaves, with frequencies ranging from 900 to 2450 MHz, generate heat by rapidly oscillating water molecules. Microwave ablation’s advantage lies in its ability to achieve higher intra-lesion temperatures and uniform heating compared with RFA. Microwave ablation’s application in pancreatic cancer and pancreatic neuroendocrine tumors has demonstrated promise with similar technical success to RFA. Yet, concern for peri-procedure complications, as well as a dearth of studies comparing RFA and microwave ablation, emphasize the need for further research. No studies have evaluated microwave ablation in PCLs. We herein review thermal ablation’s potential to treat pancreatic lesions.

## 1. Introduction

Thermal ablation is increasingly being utilized for the management of solid parenchymal tumors, such as hepatocellular cancer, renal tumors, thyroid nodules, and pulmonary tumors [1,2,3,4,5]. However, its application in the management of pancreas lesions was delayed due to fears of causing iatrogenic thermal injury to the surrounding organs [6]. The initial success of radiofrequency ablation (RFA) in inoperable pancreatic cancers led to its application in pancreatic neuroendocrine tumors and pancreatic cystic neoplasms (PCLs) [7,8,9]. Over the last decade, other techniques of thermal ablation, such as microwave ablation, high-intensity focused ultrasound, photodynamic therapy, and cryothermal ablation, have been introduced as alternatives to RFA. Amongst all modalities, microwave ablation is currently the second most common ablative technique after RFA, used in 17% of cases, and is increasingly being utilized for other types of pancreatic lesions [10]. The objective of this article, therefore, is to review the technique, advantages, and adverse events of microwave ablation for pancreatic cancer, pancreatic neuroendocrine tumors, and PCLs.

## 2. Background

Microwaves constitute a form of electromagnetic radiation characterized by a frequency ranging from 900 to 2450 MHz. When directed towards water molecules, these microwaves induce rapid oscillations, prompting water molecules to swiftly reverse their electrical charges at a rate of 2–5 billion cycles per second. This intense molecular agitation gives rise to friction and subsequent heat generation, leading to coagulation necrosis of the surrounding tissue due to the induced thermal effects [11].

In theory, compared to RFA, microwave ablation can attain elevated intra-lesion temperatures. Microwave ablation also allows concurrent application of multiple microwave antennas, facilitating more extensive and rapid tumor ablation. Furthermore, microwave ablation boasts an improved convection profile and adeptly achieves optimal tumor heating, potentially reducing procedural discomfort [11,12,13,14]. With microwave ablation, energy is emitted from a probe that is placed within a lesion or tumor. The heat generated by microwave ablation is more evenly distributed throughout the target tissue and can potentially penetrate deeper than RFA due to its higher frequency. In RFA, the heat primarily spreads outward from the ablating electrode, generating a zone of tissue injury around the electrode’s tip. The tissue in direct contact with the ablation probe experiences the highest thermal injury with immediate coagulative necrosis, whereas tissue injury due to microwave ablation is more spherical or ellipsoidal with a uniform distribution of thermal energy. From a technical perspective, microwave ablation takes less time and can be used surgically, endoscopically, or percutaneously [14,15,16].

While there have been extensive studies on RFA over the years, microwave ablation, although relatively newer, has been gaining steady traction. Both microwave ablation and RFA can be effective for various types of tumors, with the choice between the two often depending on the tumor’s location, size, proximity to critical structures, and experience of the provider. Studies comparing RFA and microwave ablation have reported similar efficacy and complication rates for hepatocellular carcinoma, parathyroid disease, pulmonary tumors, and renal tumors [16,17,18,19,20]. Microwave ablation, however, was associated with lower complication rates for the management of renal tumors and is preferred over RFA for the management of perivascular lesions and now of pancreatic lesions [16,20,21]. 

## 3. Technique

**Figure 1:**
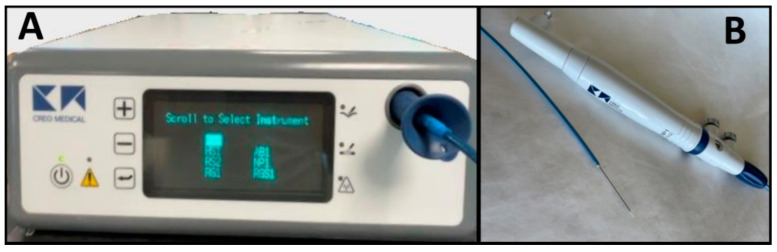
Microblate™ Fine Needle (Creo Medical, Chepstow, UK): microwave delivery system with generator (**A**) and microwave antenna needle and coaxial cable (**B**).

The microwave delivery system consists of a generator, a microwave antenna needle, and a flexible coaxial cable. In Figure 1, the EUS-guided microwave ablation probe is shown with the power generator and EUS-guided Microblate™ Fine Needle (Creo Medical, Chepstow, UK) as an example. The specific power and frequency output of the generator vary according to the manufacturer. The microwave antenna is 15 to 30 cm in length with a 14 to 20-gauge needle. The generator and antenna are connected using a flexible coaxial cable, which may be internally cooled [22].

There are multiple ways to access the pancreatic lesion for microwave ablation, including the percutaneous, laparotomy, and endoscopic ultrasound (EUS) approaches [23,24,25,26,27]. The choice of approach depends on the operator’s preference/expertise and patient characteristics. The patient’s hemodynamics should be closely monitored throughout the procedure. The postoperative course and length of hospitalization depend on the complexity of the procedure.

The general principles of performing microwave ablation of pancreatic lesions involve the following: (a) Advancing the microwave ablation probe into the lesion until its tip is situated in the center of the lesion [23,28]. When used surgically during laparotomy, a cold, wet gauze can be placed over surrounding tissue to avoid thermal damage. (b) Applying short bursts of microwave energy ranging from 60 s to 10 min at various power wattages ranging from 20 W to 60 W. The output time, power, and frequency are usually recommended by the manufacturer to allow for optimal thermal necrosis of the tissue. Multiple bursts of energy can be applied. (c) Constant monitoring of the ablation zone, which is performed using ultrasound or computed tomography imaging [24,25,29,30,31].

There is a paucity of standardized guidelines for microwave ablation due to its emergence as a novel ablative technique for pancreatic lesions. While microwave ablation is commonly employed for hepatic tumors, a comparative study utilizing two different frequencies (915 MHz vs. 2.45 GHz) demonstrated no differences in ablation outcomes. Nevertheless, variations in parameters such as total ablation time per application, ablation time per lesion, and applied energy were observed. Further research involving microwave ablation for pancreas lesions is imperative to establish the requisite technical specifics and optimal procedural details [32].

### 3.1. Radiofrequency Ablation

Numerous studies have evaluated the use of RFA in the management of pancreatic lesions [7]. RFA generates heat through a high-frequency alternative current in the range of 400–500 kHz delivered via a needle electrode to the target tissue, causing coagulative necrosis and apoptosis [33,34]. Similar to the microwave delivery system, the RFA system also consists of a generator and a needle electrode. The RFA system also consists of a grounding pad. Only one RFA system is FDA-approved in the United States: STARmed EUSRA RF (STARmed, Seoul, Republic of Korea). The device consists of an 18- or 19-gauge RFA needle, which is connected to the generator. The needle electrode is 140 cm in length and is completely covered except for the distal segment (5–20 mm in length), which delivers energy through its conical tip. This needle electrode is internally cooled with cold saline to prevent charring on the electrode surface. RFA is susceptible to the heat sink effect, where the heat is absorbed and dissipated by the bloodstream from adjacent structures, thereby reducing the ablative effect [35]. Depending on the target lesion, RFA can be applied via a percutaneous, EUS-guided, or surgical approach.

### 3.2. Photodynamic Therapy

Photodynamic therapy is a novel technique with limited clinical usage. Initial studies have reported the technical feasibility and safety of the technique in patients with inoperable pancreatic cancer [36]. During the procedure, a photosensitizer drug is administered intravenously, and multiple animal studies have shown that these photosensitizing drugs preferentially accumulate in malignant pancreatic tissue [37]. Subsequently, the target tissue is exposed to a predetermined wavelength of light that activates the photosensitized drug and causes localized necrosis. The one published study on pancreatic cancer utilized a chlorin e6 derivative (Photolon; Belmedpreparaty, Minsk, Belarus) as the photosensitizing drug [36]. Using an EUS-guided approach, they advanced a flexible laser-light catheter preloaded on a 19-gauge needle into the pancreatic tail to activate the drug and cause necrosis. Future studies should further investigate these photosensitizing drugs, the depth of penetration for various wavelengths of light, and other technical aspects of the procedure.

### 3.3. High-Intensity Focused Ultrasound

High-intensity focused ultrasound causes thermal coagulative necrosis in target tissue using a focused ultrasound beam [38]. The high-intensity focused ultrasound system consists of three main parts: I. a special transducer that bundles ultrasound waves into a beam and focuses it at the target tissue; II. a generator; and III. an imaging modality (magnetic resonance imaging or ultrasound) that targets and monitors waves in real time. Depending on the manufacturer of the system, the emitted frequency and penetrable tissue depth can differ. During the procedure, very short bursts of ultrasound energy are delivered repeatedly while the target tissue is constantly monitored via imaging modalities [39]. One benefit of a high-intensity focused ultrasound method is that it is non-invasive and does not require any needles. Many studies have reported the safety and technical feasibility of high-intensity focused ultrasound on pancreatic cancer [40]. However, more studies are needed to evaluate the use of high-intensity focused ultrasound on pancreatic neuroendocrine tumors and PCLs. 

### 3.4. Cryothermal Ablation

Similar to other thermal ablative techniques, cryothermal ablation relies on the generation of heat and subsequent irreversible cellular damage and coagulative necrosis. However, in addition to this thermal injury, cryothermal ablation induces in situ freezing, vascular injury, and apoptosis by application of a cryogenic gas such as carbon dioxide [41]. The HybridTherm Probe (Erbe Elektromedizin GmbH, Tübingen, Germany) consists of a bipolar RFA device paired with an internal carbon dioxide cooling system used under EUS guidance and has undergone limited clinical investigation [42,43]. Most recently, the system was applied to patients with locally advanced pancreatic adenocarcinoma in conjunction with standard chemotherapy versus chemotherapy alone; however, meaningful conclusions about survival could not be drawn because the study was underpowered [43]. Cryothermal ablation is thought to overcome some of the existing limitations of standard RFA; however, additional investigation is needed.

## 4. Microwave Ablation of Pancreatic Lesions

### 4.1. Pancreatic Cancer

A majority of pancreatic cancers are unresectable at the time of diagnosis [44,45]. For patients with unresectable tumors, treatment has focused on palliation and improving quality of life. Systemic chemotherapy, often combined with radiotherapy, provides limited symptomatic relief. Notably, minimally invasive, local ablative techniques have gained prominence as promising approaches. Among these options, RFA has been the mainstay for thermal ablative methods; nonetheless, complication rates have reached as high as 40% [7,46]. The comparable efficacy profile of microwave ablation, coupled with the aforementioned advantages compared to RFA, has paved the way for its integration into the management of pancreatic cancer.

In the United States, the first reported use of microwave ablation for locally advanced pancreatic cancer was in 2007 by Lygidakis et al. [26]. These authors enrolled 15 patients with locally advanced pancreatic cancer. The average size of the tumor was 6 cm (range 4–8 cm). All patients underwent microwave ablation via laparotomy. The group reported 100% technical success, and all 15 patients had partial necrosis during the 22-month follow-up. Complications included mild pancreatitis (*n* = 2), asymptomatic hyperamylasemia (*n* = 2), pancreatic ascites (*n* = 1), and minor bleeding (*n* = 1). Another case evaluating microwave ablation during laparotomy reported cyst size reduction from 43 mm to 35 mm and imaging evidence of complete ablation at 1-month follow-up. No adverse events were reported [25].

Following the technical feasibility of a laparotomy approach, Carrafiello et al. enrolled 10 patients with locally advanced pancreatic cancer (mean diameter 3.2 cm; range 2–4.3 cm) to undergo microwave ablation [23]. Half of these patients underwent percutaneous microwave ablation (*n* = 5), and the other half had laparotomy microwave ablation. The authors reported 100% technical success for both approaches. During a mean follow-up of 9.2 months (range 3–16 months), mild acute pancreatitis (*n* = 2; one complicated with pseudocyst requiring a drain) and pseudoaneurysm of gastroduodenal artery (*n* = 1) occurred in a subset of patients. This study did not provide a subgroup comparative analysis of the two modes of microwave ablation delivery, and further studies are needed. The choice should be made in the context of a multidisciplinary discussion, which considers patient anatomy and comorbidities.

Following the demonstrated feasibility of the percutaneous approach in microwave ablation, several subsequent studies have utilized this methodology. For instance, Leradi et al. conducted a study involving five patients diagnosed with locally advanced pancreatic cancer. The authors achieved a technical success rate of 100% and noted a partial response rate of 100% at the 1-month mark. Complications included asymptomatic peripancreatic fluid collection (*n* = 1) that self-resolved before discharge [24]. In another study, a percutaneous approach to microwave ablation exhibited technical success in 100% of 20 patients (across 22 sessions) with locally advanced pancreatic cancer. Notably, complications were limited to localized abdominal pain (*n* = 2). A follow-up assessment at 3 months revealed local tumor progression in 10% of patients [47].

Both the laparotomy and percutaneous approaches to microwave ablation have demonstrated technical success in the context of locally advanced pancreatic cancer. The procedure itself is well tolerated, with a cumulative complication rate of 15% observed across both approaches in the existing literature [23,24,25,26,47]. Given the technical success and relatively low complication rates, subsequent studies should evaluate the efficacy of microwave ablation for locally advanced pancreatic cancer. Studies should also evaluate the technical feasibility and efficacy of EUS-guided microwave ablation of pancreatic cancer. Future studies could explore direct comparisons between the percutaneous and laparotomy methods. Additionally, investigations comparing RFA and microwave ablation for pancreatic cancer management are essential to establish the safety and efficacy of these techniques.

### 4.2. Pancreatic Neuroendocrine Tumors

Only 10% of pancreatic neuroendocrine tumors are functional, and insulinomas are the most common among these [48]. While surgical resection has traditionally been the preferred approach for localized pancreatic neuroendocrine tumors, minimally invasive techniques are emerging as effective alternatives. Initial studies with RFA, transcatheter arterial chemoablation, chemoablation, and high-intensity focused ultrasound ablation have shown safety and efficacy in treating pancreatic neuroendocrine tumors, and these approaches are gaining popularity among surgical patients at high risk for surgery or even comorbidities associated with surgery [49,50].

Although not within the pancreas itself, the initial utilization of microwave ablation in the management of a pancreatic neuroendocrine tumor was reported in a case involving liver metastases in a patient with metastatic insulinoma. The patient had a partial pancreatectomy to remove a non-functional pancreatic neuroendocrine tumor and, years later, developed episodes of hypoglycemia and was found to have multiple liver metastases originating from the insulinoma. This patient underwent microwave ablation with transcatheter arterial chemoablation for different lesions, resulting in transient relief of hypoglycemia after each intervention [51].

Chen et al. reported the first use of microwave ablation for an insulinoma within the pancreas. A 60-year-old patient with stage IV non-small cell lung cancer and an insulinoma had recurrent admissions for hypoglycemia. The patient was a poor surgical candidate and underwent computed tomography-guided percutaneous microwave ablation with no reported complications. The patient achieved normoglycemia within 4 h of the procedure, and no further hypoglycemic episodes were recorded at 3 months follow-up [29]. In another reported case, a 3.5 cm non-functional pancreatic neuroendocrine tumor was treated by EUS-guided microwave ablation with no adverse events documented during an 8-month follow-up [27]. While interventions for small (size <2 cm) non-functional pancreatic neuroendocrine tumors are individualized, guidelines recommend resection for large pancreatic neuroendocrine tumors, and microwave ablation shows efficacy in managing patients at high surgical risk [52].

In a case series of seven patients who were either poor surgical candidates or preferred non-surgical options, subjects had either grade 1 or 2 insulinomas in the head of the pancreas (range 12–22 mm). Treatment included either percutaneous, laparotomy, or laparoscopy approaches. All patients achieved normoglycemia during an average follow-up of 31 months (range 11–42 months). There was no radiographic evidence of recurrence of the tumor. Complications included asymptomatic pseudocysts in the ablation zone (*n* = 2) and a grade B pancreatic fistula (*n* = 1) [53]. Another case report from Sri Lanka also reported post-procedural focal pancreatitis after CT-guided microwave ablation combined with alcohol ablation of an insulinoma. During a follow-up of 8 months, no further hypoglycemic episodes were documented [31].

The existing body of literature on the safety and efficacy of microwave ablation for pancreatic neuroendocrine tumors is limited, with approximately 10 cases having been evaluated. Within this limited dataset, microwave ablation has been reported to demonstrate 100% efficacy, albeit with an associated high adverse event rate. However, it is essential to underscore the need for further comprehensive studies to firmly establish the safety and efficacy profile of microwave ablation for this specific medical application. Moreover, EUS-guided microwave ablation approaches can potentially offer a more selective and precise approach, thereby minimizing adverse outcomes. 

### 4.3. Pancreatic Cystic Lesions

Intraductal papillary mucinous neoplasms (IPMNs) with multiple worrisome criteria or high-risk features represent premalignant lesions of the pancreatic epithelium with a high potential for malignant transformation to pancreatic cancer [54]. These premalignant lesions are increasingly being targeted for early diagnosis and, in some cases, prevention of pancreatic cancer [55]. Minimally invasive techniques, including EUS-guided chemoablation, thermal ablation, and cryoablation, have all been used in the management of these lesions. However, there are no reported cases of microwave ablation of PCLs to our knowledge. 

While there is currently no established evidence supporting the use of microwave ablation for PCLs, a noteworthy case involved the successful application of microwave ablation for metastatic serous cystadenoma in the liver. In this particular instance, a patient had previously undergone distal pancreatectomy for a serous cystadenoma but was later found to have metastatic serous cystadenocarcinoma in the liver. Microwave ablation was performed without any post-procedural complications, and as of the last follow-up at 3 years, there was no evidence of recurrence [30].

## 5. Discussion

Pancreatic lesions are increasingly being identified; diagnosis and risk stratification of these lesions have been challenging, but with the advent of new modalities, this is improving [55,56]. The management of these lesions is also challenging, given the suboptimal diagnosis and the morbidity/mortality of pancreatic surgeries [57,58]. Thus, minimally invasive approaches are increasingly being utilized for managing pancreatic lesions where surgery is prohibitively high-risk [59]. EUS-guided chemoablative techniques gained a lot of attention for the management of pancreatic lesions over the last two decades. Thermal ablative techniques initially showed success against non-pancreatic tumors [1,2,4,5] and were subsequently applied to pancreatic lesions over the last decade. Numerous studies have reported good outcomes with RFA against pancreatic lesions [7]. More recently, microwave ablation has been introduced and has theoretical advantages over RFA, and there are growing data on the use of microwave ablation for pancreatic and non-pancreatic lesions [60]. However, there are limited data on pancreatic cancer treatment using other thermal ablative therapies, such as high-intensity focused ultrasound, photodynamic therapy, and cryothermal ablation, which limits meaningful comparisons of these novel techniques.

Microwave ablation has many technical and procedural advantages over RFA and chemoablation (Table 1). Microwave ablation can be utilized through EUS, percutaneous, and surgical approaches. Compared to RFA, microwave ablation provides a larger ablation volume and can generate higher temperatures in a shorter duration of time [61,62,63]. Therefore, microwave ablation can be used for larger lesions with better ablation margins and is a quicker procedure. Additionally, unlike RFA, microwave ablation is not susceptible to the heat sink effect, thus lowering its potential for incomplete ablation [63].

Despite this theoretical benefit of microwave ablation over RFA, the initial clinical data on microwave ablation are divergent. Overall, there are limited studies evaluating microwave ablation in pancreatic cancer [23,24,25,26,47]. None of the microwave ablation studies utilize an EUS-guided approach. More recent studies have preferentially utilized the percutaneous approach over the laparotomy approach for microwave ablation. These factors, combined with the limited overall data, hinder comparisons with other ablative techniques, which often make use of the EUS-guided approach and have very limited data on the percutaneous or laparotomy approaches. Studies on microwave ablation have reported great technical feasibility for both the percutaneous and laparotomy approaches. Due to the heterogeneity and limited data, a subgroup analysis of complication rates for different approaches is challenging. However, the cumulative complication rate for microwave ablation is 22% (Table 2). In comparison, only one study evaluated percutaneous RFA in 35 patients with pancreatic cancer, reporting a 100% technical success rate and no complications [64]. Another study by Girelli et al. demonstrated the technical feasibility of the laparotomy approach for RFA in pancreatic cancer, reporting a 24% complication rate and a 2% 30-day mortality rate [65]. The percutaneous approach to microwave ablation offers several theoretical advantages over traditional EUS-guided RFA, primarily due to its less invasive nature. However, it is worth noting that accessing the pancreas using the percutaneous approach can be challenging.

The few studies that have evaluated microwave ablation for pancreatic neuroendocrine tumors have reported a relatively high complication rate of 36%. Interestingly, the only study evaluating EUS-guided microwave ablation reported no complications, albeit in a limited sample of only one patient [27]. The reported complication rates for the management of pancreatic neuroendocrine tumors via EUS-guided RFA and EUS-guided ethanol ablation are 14% and 17%, respectively [66,67]. With the adoption of EUS-guided microwave ablation, more meaningful comparisons of safety outcomes can be made between the different techniques. In terms of efficacy, microwave ablation resulted in 100% resolution at a 42-month follow-up, EUS-guided RFA resulted in 86% resolution at a 53-month follow-up, and EUS-guided ethanol ablation resulted in an 82% resolution at a 60-month follow-up [66,68,69]. While this is promising, it should be noted that the microwave ablation of pancreatic neuroendocrine tumors is limited to 11 cases. 

No studies have evaluated microwave ablation of PCLs or reported data on resolution rates and recurrence rates. Chemoablation has shown higher rates of complete resolution compared to RFA [70]. However, chemoablation and other injection-based techniques have limited efficacy in the management of lobulated cysts or cysts with mural nodules [71]. Thermal ablative techniques are versatile and can be used to treat a variety of pancreatic lesions, and future studies should evaluate microwave ablation of PCLs.

**Table 2:** Safety comparison of microwave ablation, radiofrequency ablation, and chemoablation for pancreatic lesions. PC—Pancreatic cancer, pNET—Pancreatic neuroendocrine tumors, PCL—Pancreatic cystic neoplasms, MWA—Microwave ablation, RFA—Radiofrequency ablation. N/A—not available. ^1^ Includes ethanol ablation of pNETs. ^2^ Includes studies with ethanol lavage followed by paclitaxel ablation of PCLs.

	Microwave Ablation Studies		Cumulative Complication Rate (%)		
	N	Complications	MWA	RFA	Chemoablation
PC	54	Abdominal pain (*n* = 2) Mild pancreatitis (*n* = 5) Asymptomatic hyperamylasemia (*n* = 1) Pancreatic ascites/fluid collection (*n* = 2) Minor bleeding (*n* = 1) Pseudoaneurysm of gastroduodenal artery (*n* = 1)	22%	27% [7]	35% [72]
pNET	11	Pseudocyst (*n* = 2) Pancreatic fistula (*n* = 1) Mild acute pancreatitis (*n* = 1)	36%	11% [7]	17% ^1^ [67]
PCL	0	N/A	N/A	20% [73]	14% ^2^ [74]

## 6. Future Directions

The initial results on microwave ablation in pancreatic lesions are promising due to the high efficacy and relatively low complication rates. Throughout this manuscript, we have identified several gaps in the existing knowledge that should be the focus of future studies. Some of these gaps are outlined in Table 3. Studies evaluating microwave ablation in the management of premalignant PCLs, such as mucinous cystic neoplasms and IPMNs, are needed. While it would be desirable to conduct studies that directly compare various thermal ablation devices, practical limitations may hinder the feasibility of such investigations due to the relatively low incidence rates of cases warranting these procedures. Therefore, future studies should prioritize assessing the feasibility and safety of thermal ablation in patients with unresectable pancreatic cancer with a specific focus on key clinical endpoints, such as overall survival and progression-free survival. There is an unmet need for well-designed comparative studies aimed at providing more comprehensive evaluations of the efficacy and safety of loco-regional ablation in carefully selected pancreatic cancer patient populations. Furthermore, it is crucial to explore the effects of varied power settings and ablation times and frequencies during microwave ablation and to conduct comparative outcome assessments in order to establish standards of care. Such investigations can significantly contribute to the standardization of microwave ablation, which will be important before more centers can adopt this technique.

## Figures and Tables

**Table 1:** Technical comparisons of chemoablation, radiofrequency ablation, and microwave ablation. RFA—Radiofrequency ablation, MWA—Microwave ablation, PC—Pancreatic cancer, pNET—Pancreatic neuroendocrine tumors, PCL—Pancreatic cystic neoplasms. N/A: Not applicable.

	Ethanol/Chemoablation	RFA	MWA
Approach			
EUS-guided	x	x	x
Percutaneous		x	x
Laparotomy		x	x
Timing of necrosis	Delayed	Immediate	Immediate
Theoretical Aspects [61,62,63]			
Ablation Volume	Dependent on cyst size	Small to medium	Large
Temperature	N/A	Low	High
Heat Sink Effect	N/A	More	Less
Need for cooling probe tip	N/A	Yes	Optional
Target Lesion	PCLs	PCLs, pNETS, PCs	pNETS, PCs PCLs (no data yet)

**Table 3:** Major gaps in the existing literature on microwave ablation of pancreatic lesions. PCLs—Pancreatic cystic lesions, EUS—Endoscopic ultrasound, pNETs—Pancreatic neuroendocrine tumors.

Gaps in Knowledge	Existing Data
Comparison of laparotomy, percutaneous, and EUS-guided approach	None
Microwave ablation in PCLs	None
Long-term efficacy data	Limited data to pNETs
EUS-guided microwave ablation	Limited data to pNETs
Effect of technical modification on efficacy and safety of microwave ablation	Data in non-pancreatic lesions

## Data Availability

No new data were created or analyzed in this study. Data sharing is not applicable to this article.
